# Osteoblast-Targeted Overexpression of TAZ Increases Bone Mass *In Vivo*

**DOI:** 10.1371/journal.pone.0056585

**Published:** 2013-02-18

**Authors:** Jae-Yeon Yang, Sun Wook Cho, Jee Hyun An, Ju Yeon Jung, Sang Wan Kim, Seong Yeon Kim, Jung Eun Kim, Chan Soo Shin

**Affiliations:** 1 Department of Internal Medicine, Seoul National University College of Medicine, Seoul, Korea; 2 Department of Molecular Medicine, Kyungpook National University School of Medicine, Daegu, Korea; Inserm U606 and University Paris Diderot, France

## Abstract

Osteoblasts are derived from mesenchymal progenitors. Differentiation to osteoblasts and adipocytes is reciprocally regulated. Transcriptional coactivator with a PDZ-binding motif (TAZ) is a transcriptional coactivator that induces differentiation of mesenchymal cells into osteoblasts while blocking differentiation into adipocytes. To investigate the role of TAZ on bone metabolism in vivo, we generated transgenic mice that overexpress TAZ under the control of the procollagen type 1 promoter (Col1-TAZ). Whole body bone mineral density (BMD) of 6- to 19-week-old Col-TAZ mice was 4% to 7% higher than that of their wild-type (WT) littermates, whereas no difference was noticed in Col.1-TAZ female mice. Microcomputed tomography analyses of proximal tibiae at 16 weeks of age demonstrated a significant increase in trabecular bone volume (26.7%) and trabecular number (26.6%) with a reciprocal decrease in trabecular spacing (14.2%) in Col1-TAZ mice compared with their WT littermates. In addition, dynamic histomorphometric analysis of the lumbar spine revealed increased mineral apposition rate (42.8%) and the serum P1NP level was also significantly increased (53%) in Col.1-TAZ mice. When primary calvaria cells were cultured in osteogenic medium, alkaline phosphatase (ALP) activity was significantly increased and adipogenesis was significantly suppressed in Col1-TAZ mice compared with their WT littermates. Quantitative real-time polymerase chain reaction analyses showed that expression of collagen type 1, bone sialoprotein, osteocalcin, ALP, osterix, and Runx2 was significantly increased in calvaria cells from Col1-TAZ mice compared to their WT littermates. In vitro, TAZ enhanced Runx2-mediated transcriptional activity while suppressing the peroxisome proliferator-activated receptor gamma signaling pathway. TAZ also enhanced transcriptional activity from 3TP-Lux, which reflects transforming growth factor-beta (TGF-β)-mediated signaling. In addition, TAZ enhanced TGF-β-dependent nuclear translocation of Smad2/3 and Smad4. Taken together, these results suggest that TAZ positively regulates bone formation in vivo, which seems to be mediated by enhancing both Runx2 and TGF-β signaling.

## Introduction

Osteoblasts are derived from mesenchymal stem cells, which are also capable of differentiating into adipocytes, chondrocytes, and myocytes. Differentiation to a particular lineage is regulated by key transcription factors; Runx2 and osterix regulate osteoblastogenesis and peroxisome proliferator-activated receptor gamma (PPARγ) and C/EBPα regulate adipogenesis [1], [2], [3], [4], [5]. Indeed, genetic ablation of Runx2 results in a complete lack of bone formation [3]. In addition, osterix null mice exhibit a similar phenotype, showing complete absence of bone formation and osteoblasts at the embryo stage although Runx2 is normally expressed [6].

Notably, there is a high degree of plasticity between osteogenic and adipogenic pathways, and differentiation to a particular lineage is accompanied by reciprocal inhibition of the alternative pathway, which is controlled by transcription factors. For instance, overexpression of PPARγ2, the key transcription factor for adipogenesis, in stromal cell lines results in suppression of Runx2 while promoting terminal differentiation to adipocytes [7], [8]. Moreover, we have shown that ectopic overexpression of PPARγ2 in MC3T3-E1 cells, determined mouse preosteoblastic cells derived from calvaria, can induce transdifferentiation of this cell line into mature adipocytes [9]. Furthermore, we also recently demonstrated that osteoblast-targeted overexpression of PPARγ results in attenuation of bone mass gain in vivo [10]. Conversely, adenovirus-mediated overexpression of Runx2 or Msx2 has strongly inhibited adipogenesis, while promoting osteoblast differentiation in mesenchymal stem cells [11] or C3H10T1/2 cells [12], respectively. These results suggest that major transcription factors play a critical role in determining the fate of mesenchymal stem cells.

TAZ is a transcriptional coactivator with a PDZ-binding motif that was discovered in a proteomic screen for 14-3-3- interacting proteins [13]. TAZ is structurally similar to a related molecule, Yes-associated protein, or YAP; both contain a 14-3-3 binding motif, single or duplicated WW domains, an extended coiled-coiled region within a large transcriptional regulatory domain, and C-terminal motif that can interact with PDZ domain-containing proteins [14]. The WW domains of TAZ and YAP bind strongly to the Pro-Pro- X-Tyr motif found within regulatory regions of Runx2 and PPARγ, and to Sox, Smad, and forkhead families [13], [15], [16]. This suggests that TAZ could play a role in the regulation of mesenchymal differentiation pathways. TAZ has been shown to interact with PPARγ and strongly inhibits adipogenesis in 3T3-L1 cells [17] whereas it enhances Runx2 transcriptional activity at the OSE2 element, the Runx2 binding site of the osteocalcin promoter [18]. In addition, a recent study has demonstrated that ERK signaling is important in the induction of osteoblast differentiation by TAZ [19]. Moreover, depletion of TAZ in zebrafish has resulted in impaired bone development and a lack of skeletal ossification [16]. These results indicate that TAZ acts as an essential transcriptional modifier of mesenchymal stem cell differentiation by promoting osteogenic differentiation while suppressing adipogenic differentiation, thus functions as a molecular switch to direct mesenchymal stem cell differentiation pathway. However, in spite of this critical role of TAZ in mesenchymal differentiation, survivors of TAZ null mice have only minor skeletal phenotype alterations [20]. Therefore, this study was undertaken to investigate the function of TAZ in the regulation of bone mass in vivo using mice model that overexpress TAZ in osteoblasts driven by procollagen type 1 promoter. We also wanted to analyze the cellular mechanisms involved in the TAZ-mediated signaling.

## Materials and Methods

### Construct

**Figure 1:**
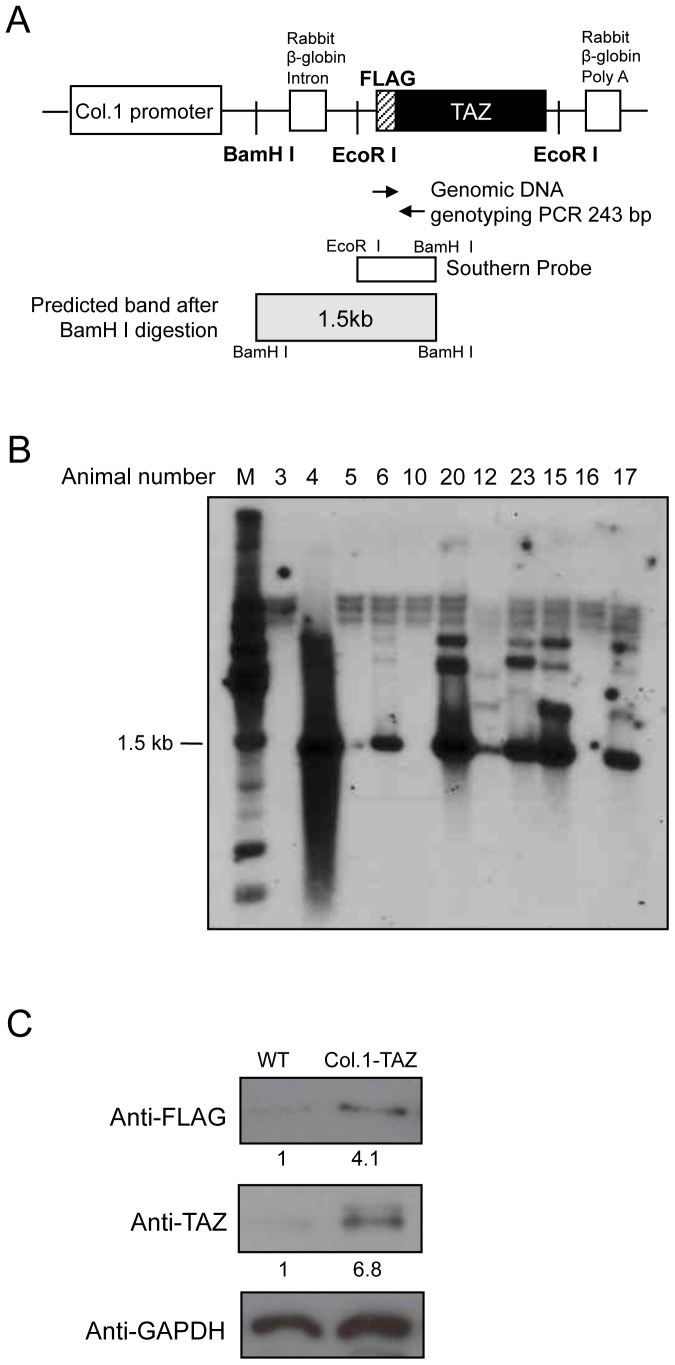
Development of Col.1-TAZ transgenic mice. (A) Linearized structure of the Col.1–TAZ construct used for transgenic expression. The coding sequence of mouse TAZ was placed under the pro-α1 (I) collagen promoter. The 350 bp EcoRI-BamHI fragment was used as a probe in Southern blot analysis. The two-way arrows indicate the primers used for PCR genotyping. Both DNA probe and the 243 bp PCR product encompass the junction between the Flag tag and the 5′end of the TAZ reading frame. (B) Southern blot of the genomic DNA of a litter showing a 1.5 kb band product corresponding to the transgene. (C) Total protein extracts isolated from calvariae of either Col.1–TAZ mice or the WT littermate were separated by SDS-PAGE and blotted (Western blot) using an anti-Flag or anti-TAZ antibody. A band of 50 kDa, corresponding to the transgene, was detected by an anti-Flag antibody in the extracts from Col.1–TAZ mice but not from the WT littermate. Expression of the transgene was further confirmed by increased expression levels of TAZ in Col.1–TAZ mice detected by the TAZ antibody compared to the WT littermate.

An expression vector for TAZ (pEF-Flag-TAZ) was obtained from Prof. Jung Ho Hong (Korea University, Seoul, Korea). We subcloned the 2.3 kb osteoblast-specific promoter region of mouse pro-α1 (I) collagen (kindly provided by Dr. Gerald Karsenty, Columbia University) into the Not I, Bam HI site of a pBluescript plasmid, resulting in a pBluescript-Col I plasmid. We subcloned a rabbit β-globin intron, polyA of pSG-2 (kindly provided by Dr. Jun-ichi Miyazaki, Osaka University, Suita, Japan) into the BamHI, SalI site of our pBluescript-Col I plasmid. A 1-kb EcoRI fragment of the pEF-Flag-TAZ vector was ligated into the pBluescript-Col I- rabbit β-globin construct, resulting in the pBS-Flag-TAZ recombinant plasmid. This new recombinant plasmid was digested with KpnI and the 3.5 kb DNA fragment was purified by agarose gel electrophoresis for microinjection (Fig. 1A). pcDNA3-til, an expression vector for type II isoform of Runx2, which starts in exon 2 and has an N-terminal extension of 19 amino acids [21], was kindly provided by prof. Je-Yong Choi (Kyungpook National University, Korea).

### Generation of transgenic mice and determination of transgene expression

All animal procedures were reviewed and approved by the Seoul National University Institute of Laboratory Animal Resources (approval #SNU-110620-5). Transgenic mice were generated using a standard procedure at the Center for Animal Resource and Development, Seoul National University College of Medicine. The fertilized one-cell embryos were obtained from 4-week-old superovulated C57BL/6J females. Purified DNA fragments were microinjected into the pronuclei of embryos. Microinjected eggs were implanted into oviducts of pseudopregnant female mice and carried to term. Twenty-four founders from 5 litters were screened for the presence of the transgene by polymerase chain reaction (PCR) amplification of genomic DNA isolated from the tail.

The PCR primers were designed to amplify a sequence 5′-coding region of the TAZ gene yielding a 300 bp product (sense 5′-TGCTGTCTCATCATTTTGGC-3′, antisense 5′-GGGCTCCTTAAAGAAGGACT-3′; Fig. 1A). The reaction was performed with pre-denaturation at 95°C for 3 min, denaturation at 94°C for 30 sec, annealing at 52°C for 30 sec, and extension at 72°C for 30 sec for 30 cycles. Positive founders for TAZ were also identified by Southern blotting (Fig. 1B). High-expressing male mice were selected and crossed with wild-type (WT) littermate female mice.

### Measurement of bone mineral density and microcomputed tomography (μCT) analysis

To measure the bone mineral density (g/cm^2^) of the whole body, we used a dual energy X-ray absorptiometry (DXA) instrument, the PIXIMUS (GE Lunar, Madison, WI), and excluded the head region. The animals were placed in a prone position under anesthesia induced by subcutaneous injections of xylazine (2.2 mg/kg; Rompun, Bayer, Monheim, Germany) and tiletamine/zolazepam (6.0 mg/kg Zoletil 100; Virbac, Carros Cedex, France). A phantom was scanned daily for quality control. Radiographic images of the total skeletons were obtained using a small animal X-ray at 36 kV for 1 min (Faxitron X-ray Co., Buffalo Grove, IL).

For three-dimensional measurements with microCT, tibia obtained from 20-week-old male mice were dissected free of soft tissue, fixed in 70% ethanol for 24 hrs, and analyzed with a microCT scanner and the associated analysis software (model 1076, Skyscan, Antwerp, Belgium) at a 9-µm voxel size. Image acquisition was performed at 100 kV(35 KV) energy and 100 µA (220) intensity. Thresholds were applied to the images to segment the bone from the background; the same threshold settings were used for all samples. Two-dimensional images were used to generate three-dimensional reconstructions using the 3D Creator software supplied with the instrument.

### Mineralized bone histology and bone morphometry

All histological analyses were performed using 12-week-old transgenic (Col.1-TAZ) and WT littermate male mice. Mice were injected with calcein (30 mg/kg body weight) intraperitoneally at 6 and 1 day prior to sacrifice. Whole spines were isolated and fixed in 4% paraformaldehyde at 4°C overnight. The undecalcified vertebrae were dehydrated, cleared in xylene, and embedded in methyl methacrylate [22]. Next, 5-µm lumbar vertebrae sections were stained with van Gieson and von Kossa reagents. We confirmed calcein double labeling by fluorescence microscope. Static and dynamic histomorphometric analyses were conducted on the lumbar vertebrae using the Bioquant program (Bio-Quant. Inc., San Diego, CA) [23]. For evaluating the number of osteoclasts, decalcified long bones were embedded in paraffin. Serial sections were prepared from paraffin blocks at 6-µm thicknesses and were stained for tartrate resistant acid phosphatase (TRAP) activity, with 1% methyl green as the counterstain.

### Calvaria cell culture

Mouse neonatal calvaria cells were isolated from 4-day-old mouse pups. Briefly, the animals were sacrificed by decapitation and the calvariae were removed, washed 1× with Hank's Balanced Salt Solution (Invitrogen, Grand Island, NY), and bathed in alpha-minimum essential medium (αMEM; Invitrogen) containing 100 U/ml penicillin/100 µg/ml streptomycin (Invitrogen). After trimming off excess soft tissue, calvariae were transferred to αMEM containing 0.03% collagenase and 0.2% dispase and immersed in an oscillating 37°C water bath for digestion. The first 10-min digests were discarded and the second to fifth 15-min digests were harvested and filtered through a 70-µm nylon filter (Falcon, Franklin Lakes, NJ). The filtrates were centrifuged for 5 min at 1500 rpm, the supernatant was removed, and cells were plated at 5×10^4^ cells/ml into a p60 dish. The first attached cells were designated “passage 1 (P1).” Confluent calvaria cells were passaged further and plated at a dilution of 1∶2 to 1∶3. P3 or P4 calvaria cells were used for differentiation.

### Osteogenic differentiation and alkaline phosphatase (ALP) assay

The isolated calvaria cells were seeded into 24-well plates at a density of 1×10^4^ cells per well and grown to 90% confluence for 2 days. Osteogenic differentiation was induced by replacing the medium with osteogenic medium containing L-ascorbic acid (50 µg/ml) and β-glycerophosphate (10 mM). Cultures were maintained for 6 days. To assess ALP activity, the cells were washed with phosphate buffered saline (PBS) (pH 7.4) and 0.5% triton X-100 was added. Enzyme activity assays were performed in an assay buffer (10 mM MgCl_2_ and 0.1 M alkaline buffer, pH 10.3) containing 10 mM p-nitrophenylphosphate as a substrate. Absorbencies were read at OD405. Relative ALP activity is defined as millimoles of p-nitrophenyl phosphate hydrolyzed per minute per milligram of total protein. For ALP staining, cells were fixed with 3.7% formaldehyde and incubated with ALP staining solution (10 mM MgCl_2_ and 0.15 M NaCl, pH 8.8) containing the 5-bromo-4-chloro-3-indolyl-phosphate/nitro-blue tetrazolium color development substrate for 1 hr.

In experiments that assessed the effects of TGF-β inhibitors, SB-431542 and SB-525334 (Selleckchem, Houston, USA), the C3H10T1/2 cells were cultured in osteogenic medium in the absence or presence of SB-431542 or SB-525334 for 8 days and osteogenic differentiation was assessed by measuring ALP activities.

### Adipogenic differentiation and Oil-red O stain

The isolated calvaria cells were seeded in 12-well plates at a density of 2×10^4^cells per well and grown for 5–6 days to 100% confluences. Adipogenic medium containing insulin (10 µg/ml), isobutylmethylxanthine (0.2 mM), and dexamethasone (0.1 µM) was added and the cultures were maintained for 14 days. For Oil-red O staining, cells were fixed with 3.7% formaldehyde for 15 min at room temperature and rinsed in distilled water. A working stock solution of Oil Red O was prepared from 1% (wt/vol) Oil Red O in 99% isopropanol and diluted to 0.4% (vol/vol) with distilled water. Cultures were stained with working solution for 60 min and rinsed with distilled water.

### Bone marrow macrophage (BMM) culture and osteoclastogenesis

Bone marrow cells were obtained from the long bones of 8-week-old mice. Bone marrow cells were cultured in with 30 ng/ml rmM-CSF (R&D Systems, Minneapolis, MN, USA) for 3 days to generate the bone marrow–derived macrophages (BMMs). To examine osteoclast formation, BMMs were plated into 96-well culture plates (1.5×10^4^ cells/well) and cultured with 30 ng/ml rmM-CSF and 100 ng/ml mRANKL (PeproTech Inc., Rocky Hill, NJ, USA). After 4∼5 day, cells were fixed and stained for tartrate resistant acid phosphatase (TRAP), a marker enzyme of osteoclasts.

### Reverse-transcriptional PCR

First-strand cDNA was synthesized from 2 µg total RNA using a Reverse Transcription System kit (Promega, Madison, WI). The forward and reverse primer sequences used to amplify marker genes for osteogenesis and adipogenesis are listed in Table 1. Quantitative PCR was performed by SYBR Green PCR technology with an ABI PRISM 7900 HT sequence detection system (Applied Biosystems, Carlsbad, CA). Reactions were performed at thermal cycling conditions of 10 sec at 95°C, 40 cycles of 30 sec at 95°C, 30 sec at 56°C, 30 sec at 72°C. Relative gene expression was measured by comparing ratios to the β-actin cycle threshold. All PCRs were performed in triplicate.

**Table 1:** PCR primer sequences used to amplify each of the genes by RT-PCR.

Gene	Primer	Sequences (5′→3′)	Product size (bp)
Runx2	Forward	CTGTGGTAACCGTCATGGCC	49
	Reverse	GGAGCTCGGCGGAGTAGTTC	
Osterix	Forward	ATTGAATTTGGAGGAATGGT	217
	Reverse	CTTGAAGTACGTGTAACGTG	
Col1	Forward	GCGAAGGCAACAGTCGCT	101
	Reverse	CTTGGTGGTTTTGTATTCGATGAC	
ALP	Forward	TCAGGGCAATGAGGTCACATC	68
	Reverse	CACAATGCCCACGGACTTC	
Osteocalcin	Forward	CCACCCGGGAGCAGTGT	214
	Reverse	GAGCTGCTGTGACATCCATACTTG	
aP2	Forward	GTCACCATCCGGTCAGAGAGTAC	86
	Reverse	TCGTCTGCGGTGATTTCATC	
Adipsin	Forward	GCTATCCCAGAATGCCTCGTT	71
	Reverse	CCACTTCTTTGTCCTCGTATTGC	
LPL	Forward	AAGGTCAGAGCCAAGAGAAGCA	98
	Reverse	CCAGAAAAGTGAATCTTGACTTGGT	
RANKL	Forward	TGGAAGGCTCATGGTTGGAT	75
	Reverse	CATTGATGGTGAGGTGTGCAA	
OPG	Forward	AGCTGCTGAAGCTGTGGAA	115
	Reverse	TGTTCGAGTGGCCGAGAT	
β-actin	Forward	TGGGTATGGAATCCTGTGGC	103
	Reverse	CCAGACAGCACTGTGTTGGC	

Col1: type 1 collagen, ALP: alkaline phosphatase, LPL: lipoprotein lipase.

### Western Blot analysis

Cells were lysed in 50 mM Tris (pH 7.5), 150 mM NaCl, and 1% Triton X-100 supplemented with a protease inhibitor and phosphatase inhibitor mixture (Sigma, St. Louis, MO; each added at a dilution of 1∶100). Lysates were size-separated by sodium dodecyl sulfate polyacrylamide gel electrophoresis (SDS-PAGE) and transferred to a PVDF membrane (Millipore, Billerica, MA). Blots were blocked with 5% milk for 1 hr at 4°C, incubated with appropriate primary and secondary antibodies for 24 hrs and 1 hr, respectively. Blots were washed (three times for 20 min each) and proteins were visualized using an enhanced chemiluminescence kit (Amersham, Piscataway, NJ).

For the cellular localization of proteins, nuclear extracts from the cells were isolated using a Qproteome Nuclear Protein Kit (Qiagen, Valencia, CA). Proteins were analyzed by immunoblotting with rabbit anti-Smad2/3 (1∶1000, Cell Signaling Technology, Danvers, MA), mouse anti-Smad4 (1∶1000, Santa Cruz Biotechnology, Santa Cruz, CA), mouse anti-FLAG (1∶1000, Sigma), a cytoplasmic control anti-Calnexin (1∶1000, Cell Signaling Technologies, Danvers, MA), and mouse anti-Actin (1∶1000, Santa Cruz Biotechnology) antibodies.

### Transfections and reporter assays

The TOPflash reporter construct was kindly provided by Dr. Roberto Civitelli (Washington University, St. Louis, MO). Dr. Jung Tae Koh (Chonnam National University, Kwangju, Korea) kindly provided a bone morphogenic protein (BMP)-responsive reporter (BRE-Luc). Dr. Patricia Ducy (Columbia University, New York, NY) kindly provided a 1.3-kb murine osteocalcin promoter that contains one binding site for Runx2 (-1.3OG2-Luc) and p6OSE2-Luc that contains six copies of OSE sequences of the mouse osteocalcin promoter. We purchased p3TP–Lux, which contains a TGF-β response element, from Addgene (Cambridge, MA).

Mouse embryonic mesenchymal C3H10T1/2 cells or stromal/osteoblastic UAMS-32 cells were seeded into 24-well plates at 50,000 cells/well, and 12 hrs later, reporter vectors were transiently transfected into the cells. As positive controls, Wnt-3A CM (0.5×), BMP-2 (100 ng/ml), or TGF-β (5 nM) were added to cells transfected with TOPflash, BRE-Luc, or p3TP-Lux, respectively. For the p6OSE2-Luc reporter, 400-ng/well of pcDNA3-til was cotransfected. Cell lysates were collected using the Dual-luciferase assay system (Promega), and luciferase activity was measured with a luminometer (Lumat LB 9507, Berthold, Germany). An internal standard, pRL-TK *renilla* luciferase control vector (Promega), was cotransfected to normalize for transfection efficiency. All luciferase activity values were normalized to *renilla* luciferase activity and expressed as fold induction relative to basal promoter activity.

### Immunofluorescence microscopy

Cells were washed with PBS, fixed with 4% paraformaldehyde, and permeabilized with 100% methanol prior to addition of the appropriate primary and secondary antibodies. Primary antibodies included rabbit anti-TAZ (1∶100; Abcam, Cambridge, MA), mouse anti-Smad2/3 (1∶500; BD Transduction Laboratories), and mouse anti-Smad4 (B8, 1∶250; Santa Cruz Biotechnology). Secondary antibodies (1∶500; Molecular Probes, Grand Island, NY) included Alexa Fluor 488, 594 goat anti-Mouse, and Rabbit IgG. Cell nuclei were stained with DAPI dye. Fluorescence analyses were performed with a spinning disk confocal microscope (Carl Zeiss, Jena, Germany) using the Target Activation assay algorithm as part of the Cellomics Arrayscan VTI platform (Cellomics, Pittsburg, PA).

### Chromatin immunoprecipitation (ChIP)

ChIP was performed using the EZ-ChIP (Millipore) kit. The isolated calvaria cells were differentiated by treatment with 150 ng/ml rhBMP-2 (R&D Systems) for 2 days and harvested for analysis. Immunoprecipitations were carried out using the mouse anti-FLAG antibody and primers for analysis were designed to amplify the osteocalcin binding element; forward primer 5′-CTGAACTGGGCAAATGAGGACA-3′, reverse primer 5′-AGGGGATGCTGCCAGGACTAAT-3′.

### Bone turnover markers

The blood of Col.1-TAZ mice and their WT littermates at 8 weeks old were harvested by cardiac puncture and centrifuged immediately to extract serum. The serum levels of procollagen type 1 N-terminal propeptide (P1NP; Immunodiagnostic Systems Inc, AZ) were determined by enzyme-linked immunosorbent assay.

### Statistical analysis

All data are expressed as mean ± standard deviation (SD). The statistical significance of differences between the groups at each time-point was determined by a non-parametric Kruskal-Wallis test. A *p*<0.05 is considered statistically significant. Cell culture experiments were performed in triplicate (or more) and repeated twice. For static and dynamic histomorphometry, more than three animals per group were used.

## Results

### Generation of TAZ transgenic mice and examination of transgene expression

We obtained 24 founders from 5 litters and screened for the presence of the transgene by PCR amplification of genomic DNA isolated from the tail. Six pups were positive for the TAZ gene. This was further confirmed by Southern blot analysis, as shown in Fig. 1B. The three high-expression male mice were established and maintained. However, studies reported here are from a single line (line number 20), which was determined to have the highest transgene copy number. The lines were continuously back-crossed into wild type littermate female mice. Our analyses were performed on transgenic mice (Col.1- TAZ) in the fourth to sixth generations using WT littermate mice as controls. The transgene was transmitted to the progeny at the expected Mendelian frequency.

The Flag epitope, linked to the transgene, was detected by Western blot analysis of primary calcarial cells using an anti-Flag antibody (Fig. 1C). TAZ protein expression detected by anti-TAZ was also significantly increased in transgenic mice compared with WT littermates.

### Overexpression of TAZ increases bone mass

**Figure 2:**
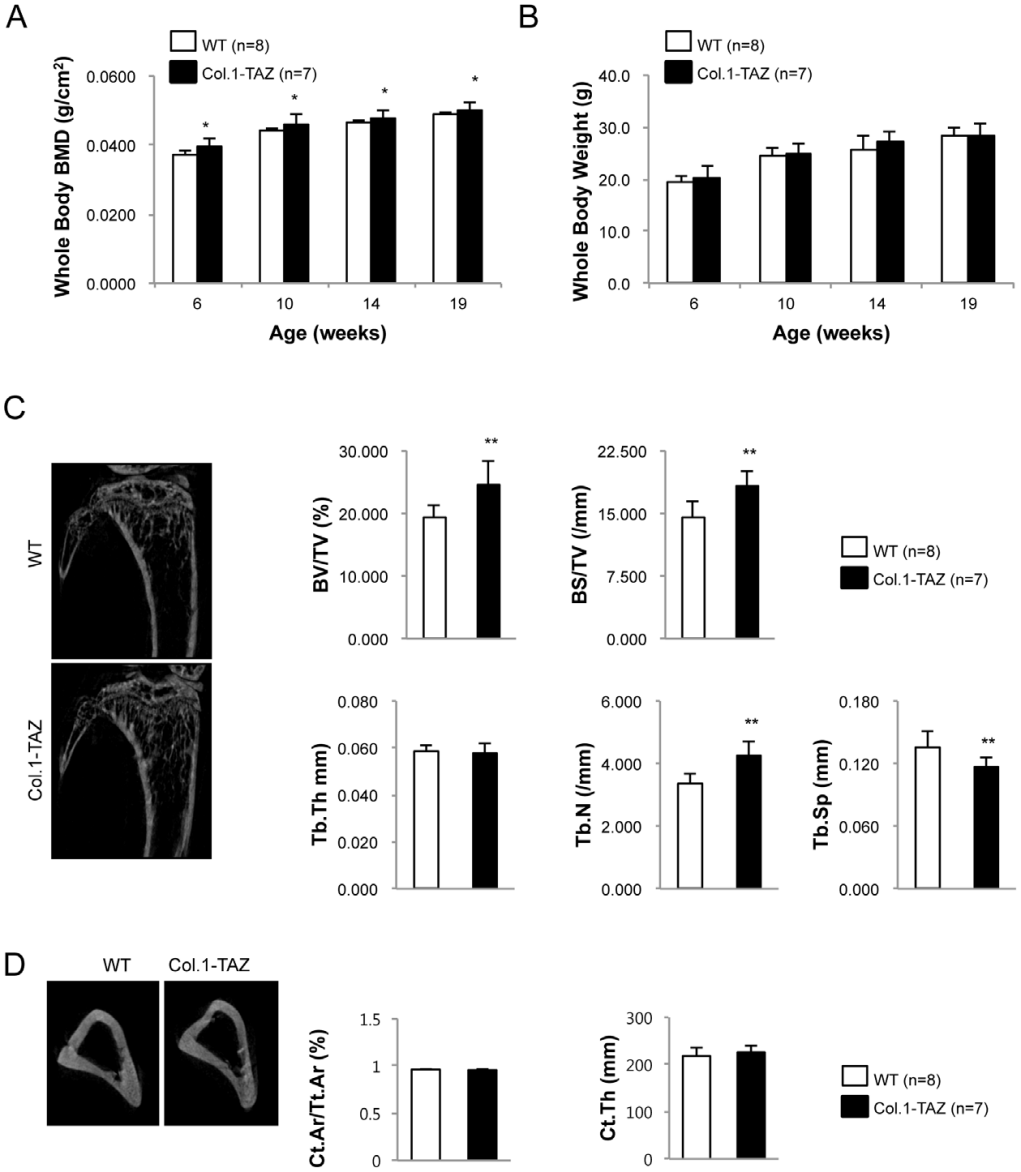
Body weight and bone phenotype in Col.1–TAZ mice. Total body BMD (A) was analyzed by dual energy X-ray absorptiometry (PixiMus). Body weight (B) was measured in male Col.1– TAZ mice and their WT littermates at 6, 10, 14, and 19 weeks (* p<0.05 vs. WT). (C) Representative μCT images of the proximal tibia from a 20-week-old male Col.1–TAZ mouse and its WT littermate. Static morphometric parameters including trabecular bone volume expressed as percentage of total tissue volume (BV/TV), trabecular number (Tb.N), trabecular thickness (Tb.Th), and trabecular spacing (Tb.Sp) were measured with the CT images. (D) Representative μCT images of the mid-femur from a 20-week-old male Col.1–TAZ mouse and its WT littermate. Cortical histomorphometric parameters were analyzed for cortical bone area (Ct.Ar) and average cortical thickness (Ct.Th) (* p<0.05 vs. WT littermates). Data are expressed as means ± SD.

Col.1–TAZ and WT littermates of both genders were weighed and body composition measured by DXA at 6, 10, 14, and 19 weeks of age. BMD analysis using DXA demonstrated that the BMD of male Col.1-TAZ mice was 4∼7% higher compared with WT littermates from 6 to 19 weeks of age (Fig. 2A). However, there was no significant difference in body weight (Fig. 2B) or total body fat content (data not shown) between Col.1–TAZ and WT mice.

Consistent with the DXA results, μCT analyses of tibiae at 20 weeks of age also showed an increase in bone volume (BV/TV) by 26.7% (*p*<0.01), bone surface density (BS/TV) by 25.1% (p<0.05), and trabecular number (Tb.N) by 27.6% (*p*<0.01). In contrast, there was a reciprocal reduction in trabecular spacing (Tb.Sp) by 14.2% (*p*<0.01) in Col.1-TAZ mice compared to their WT littermates (Fig. 2C). However, analysis of cortical bone at the mid-diaphysis of the femur showed no significant changes in cortical bone area or average cortical thickness (Fig. 2D). A second founder line of Col.1-TAZ mice (line number 23 in Fg. 1A) was investigated to confirm these findings up to 32 weeks of age. Similar to the findings in the line 20, male mice in this second line evidenced a greater BMD than did the WT animals from 8 to 32 weeks of age (5∼11%; P<0.05 at 12, 20 and 32 weeks p<0.01 at 8 and 16 weeks). In addition, trabecular parameters at 32 weeks of age also showed comparable changes as in line 20. Furthermore, cortical bone area (Ct.Ar/Tt.Ar, 14.3%, p<0.05) and cortical thickness (Ct.Th, 20.8%, p<0.05) were also increased in this line (Fig. S1).

In contrast to the pronounced bone phenotype in male mice, there was no significant difference in the BMD or body weight in female Col.1-TAZ mice compared to the WT up to 32 weeks in age (data not shown). In addition, μCT analyses of femurs did not show a significant difference in trabecular or cortical parameters between female Col.1-TAZ mice and WT littermates (data not shown). Finally, adipocytes in the bone marrow space of the distal femur were not significantly different in size or numbers between Col.1-TAZ mice and their WT littermate (data not shown).

### Overexpression of TAZ in osteoblasts increases bone formation rates

**Figure 3:**
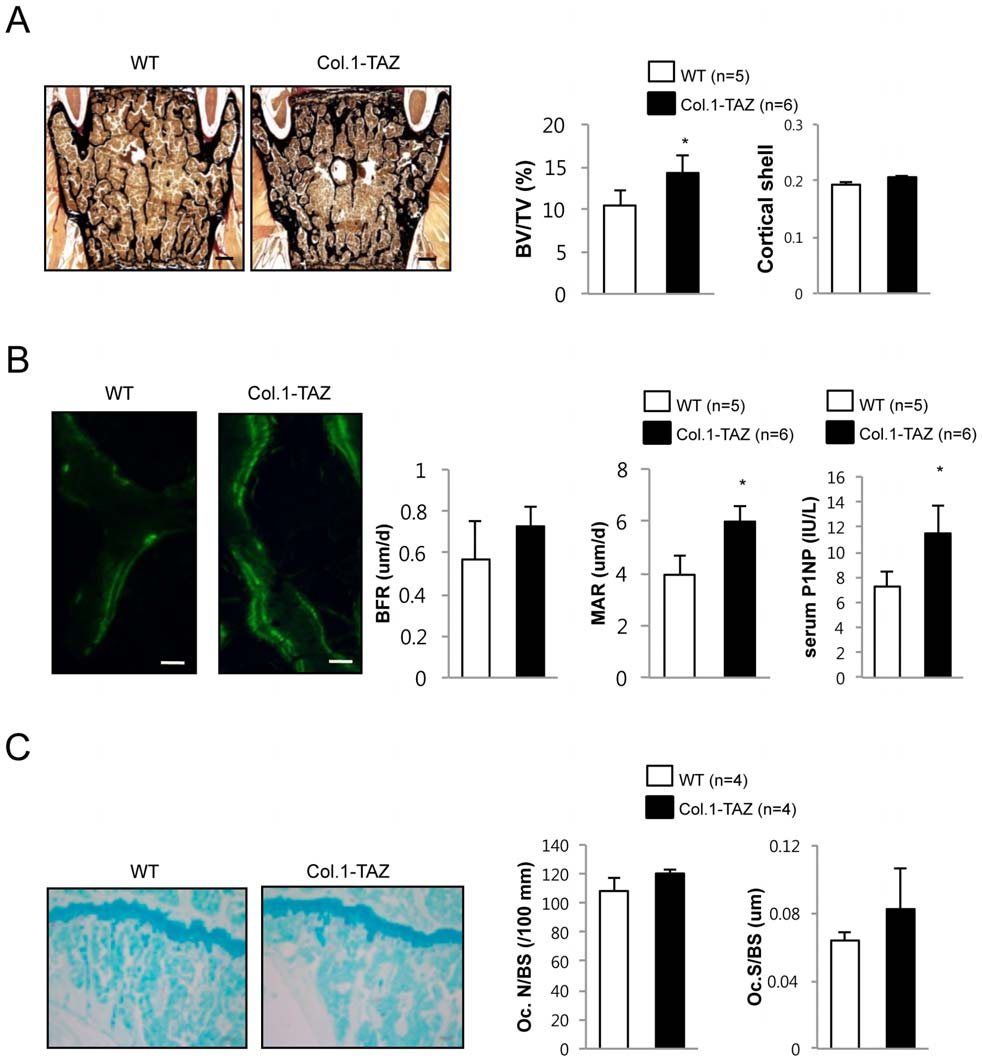
Histological analyses of male Col.1–TAZ mice and WT littermates at 20 weeks of age. (A) Representative von Kossa staining images of an undecalcified section of the lumbar spine (scale bar: 100 µm) and histomorphometric measurements for BV/TV and cortical shell thickness (Ct. Shell Th.). (B) Fluorescent images obtained from lumbar spine (scale bar: 100 µm) after calcein double labeling. Dynamic histomorphometric analyses of lumbar spine including bone formation rate (BFR) and mineral apposition rate (MAR). Serum levels of P1NP, a bone formation marker, at 8 weeks of age. (C) Representative TRAP staining images of a decalcified bone section from the distal femur (scale bar: 50 µm). Histomorphometric analyses of TRAP-stained bone sections including osteoclast number (Oc.N/BS) and osteoclast surface (Oc.S/BS). Data are expressed as means ± SD (* p<0.05 vs. WT littermates).

Next, we investigated skeletal phenotypes of the lumbar vertebrae using 20-week-old male mice. Consistent with μCT analysis of the tibiae, the histomorphometric analysis of the 4^th^ lumbar vertebrae stained with von Kossa demonstrated a significant increase in BV/TV (20.9%, p<0.05, Fig. 3A) although cortical shell thickness of the vertebrae was not significantly different between the two groups (Fig. 3A), as was the case in femur mentioned above. Calcein double labeling, which permitted an assessment of dynamic parameters of bone formation showed that mineral apposition rate (42.8%, p<0.05) was significantly increased in Col.1-TAZ mice compared with their WT littermates (Fig. 3B) although the increase in bone formation rate did not reach statistical significance. Furthermore, consistent with histomorphometric parameters, serum levels of P1NP, a biochemical marker of bone formation, were significantly increased by 53% (p<0.05) in Col.1-TAZ mice. However, when we examined the number of osteoclasts in the femurs, we found no significant difference in TRAP-positive osteoclasts (Oc.N/BS) or the osteoclast surface (Oc.S/BS) between Col.1-TAZ and their WT littermates, suggesting that osteoblast-specific overexpression of TAZ does not affect osteoclast formation in vivo (Fig. 3C).

### TAZ overexpression leads to increased osteoblastogenesis but does not affect osteoclastogenesis *in vitro*

**Figure 4:**
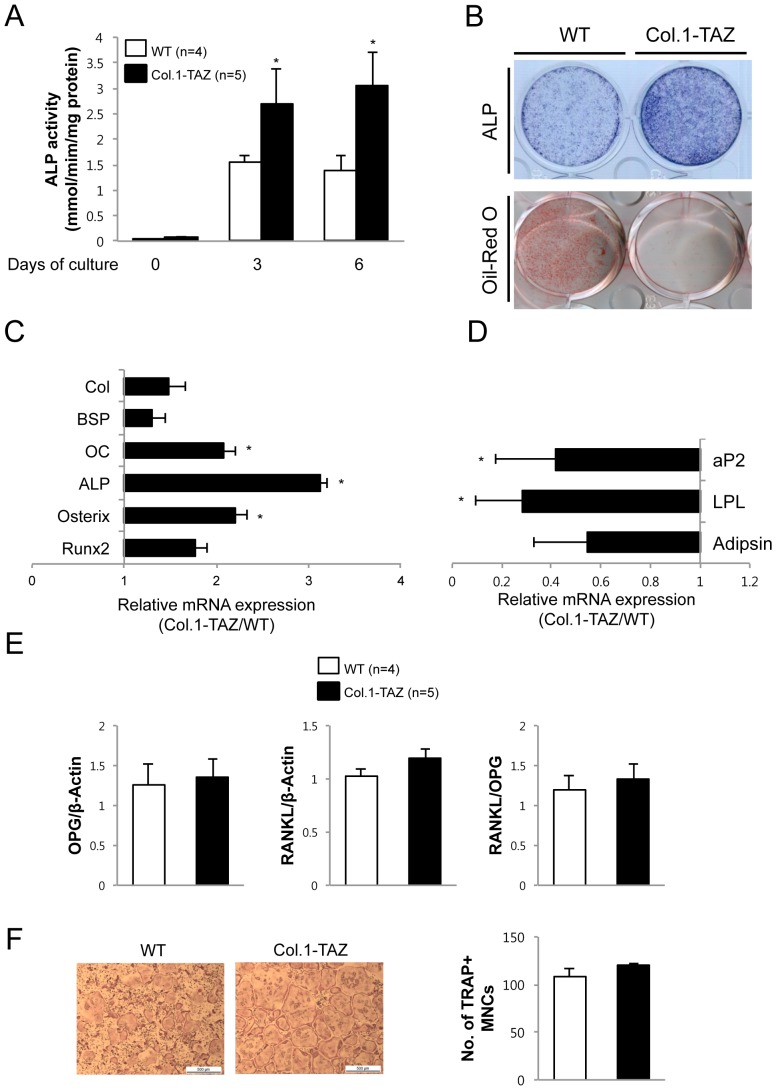
TAZ overexpression enhances osteoblast differentiation but does not affect osteoclast formation in vitro. (A) Calvarial cells from Col.1–TAZ mice or their wild-type littermates (WT) were cultured for 6 days in osteogenic medium (OM) including ascorbic acid and β-glycerophosphate. ALP activity was measured in the cell layer and normalized to cellular protein content. Data are expressed as means ± SD (* p<0.05 vs. WT) (B) The same sets of cells were cultured for 10 days in adipogenic medium containing 10 µg/ml insulin, 0.1 µM dexamethasone, and 0.2 mM 3-isobutyl-1-methylxanthine (IBMX), and stained with Oil Red O. (C) Total RNA was extracted from calvarial cells grown for 7 days in OM. The amount of mRNA for osteoblast marker gene was determined by real-time quantitative PCR and expressed as mRNA abundance in Col.1–TAZ mice relative to their WT littermates. (D) Total RNA was extracted from calvarial cells grown for 10 days in adipogenic medium. The amount of mRNA for adipogenic markers was determined by real-time quantitative PCR and expressed as mRNA abundance in Col.1–TAZ mice relative to their WT littermates. Data are expressed as means ± SD. * p<0.05 vs. WT (E) Quantitative RT-PCR analysis of RANKL and OPG gene expression and the ratio of RANKL∶OPG gene expression in calvarial cells. Data are expressed as means ± SD. (F) Representative TRAP-stained osteoclasts and the number of TRAP-positive multinucleated cells after 4∼5-day cultures of bone marrow macrophages (BMM) isolated from 6∼8-week old Col.1–TAZ mice and WT littermates in the presence of RANKL and M-CSF. Scale bar = 500 µm.

We performed ex vivo culture of calvarial cells isolated from Col.1-TAZ mice or their WT littermates. During culture with osteogenic medium that included L-ascorbic acid and β-glycerophosphate, neonatal calvarial cells from Col.1-TAZ mice showed an 87 and 114% increase in ALP activity at day 3 and 6, respectively, compared to cells from the WT littermates (Fig. 4A and B upper panel). Conversely, when we treated the calvarial cells with insulin, dexamethasone, and 3-isobutyl-1-methylxanthine (IBMX) to induce adipogenic differentiation, the degree of adipogenesis was significantly decreased in cells from Col.1-TAZ mice compared to those from WT littermates (Fig. 4B lower panel). These phenotypic changes were accompanied by corresponding changes in the mRNA expression of key markers for osteoblastogenesis and adipogenesis. As shown in Fig. 4C, mRNA expression of collagen type 1 (Col1), bone sialoprotein, osteocalcin, ALP, osterix, and Runx2 in calvarial cells from Col.1-TAZ mice was significantly increased compared to that from WT littermates. In contrast, during adipogenic differentiation, the expression levels of aP2, lipoprotein lipase (LPL), and adipsin were significantly down-regulated in cells from Col.1-TAZ mice compared to cells from the WT littermates (Fig. 4D). However, when we measured the mRNA expression of RANKL, OPG, and the RANKL/OPG ratio, no significant difference was observed between Col.1-TAZ mice and their WT littermates (Fig. 4E). Furthemore, culture of BMMs in the presence of RANKL did not result in significant difference in the number of osteoclasts generated from BMMs of Col.1-TAZ or WT mice (Fig. 4F).

### Overexpression of TAZ enhances Runx2 and TGF-β signaling

**Figure 5:**
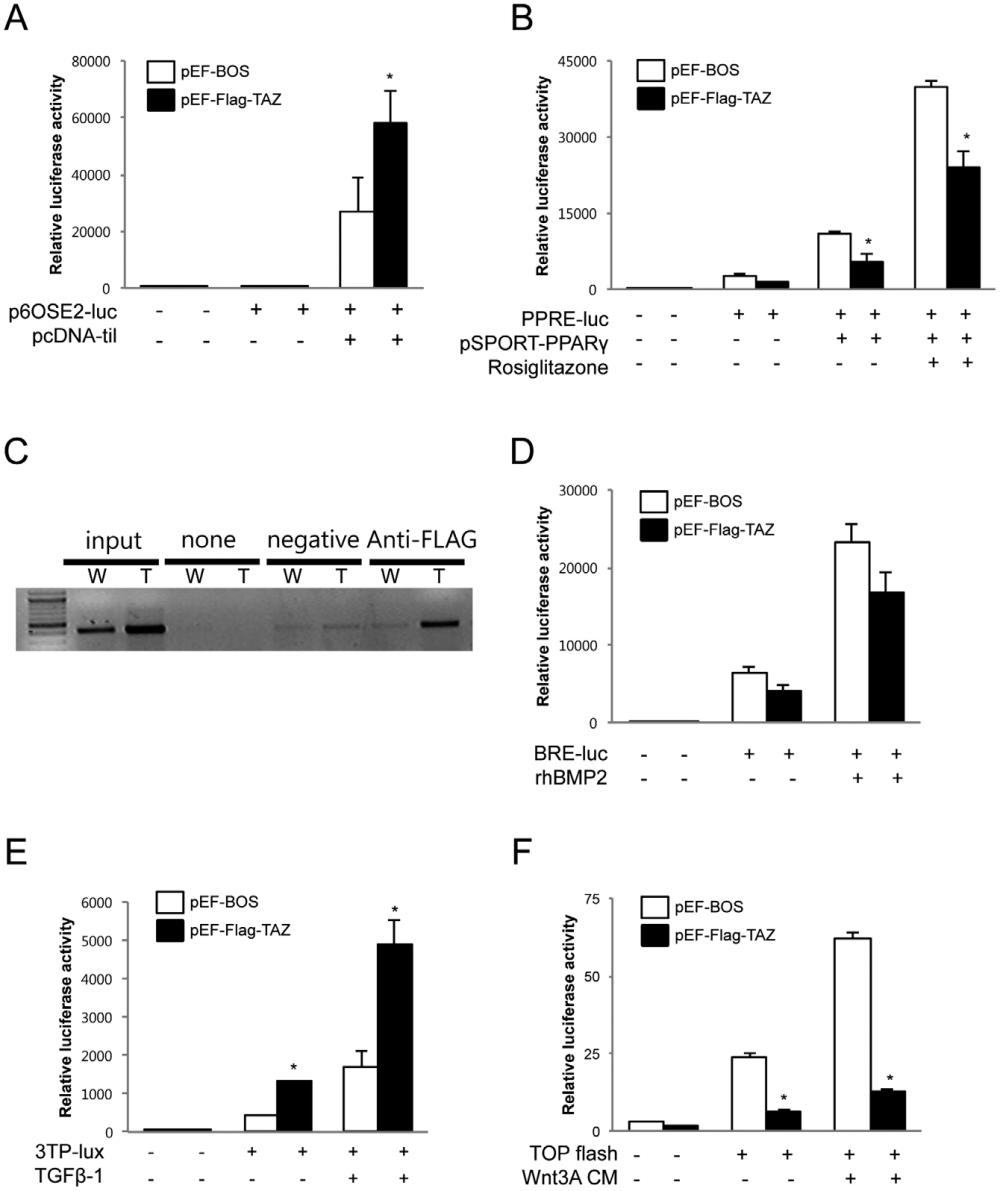
Regulation of signaling pathways by TAZ in vitro. (A) C3H10T1/2 cells were transiently cotransfected with the p6OSE2-Luc plasmid and expression vector for TAZ (pEF-Flag-TAZ) or with the empty vector (pEF-BOS). The cells were also transfected with the expression vector for til (pcDNA3-til) or empty vector as indicated. (B) C3H10T1/2 cells transiently cotransfected with PPRE-Luc and the expression vector for TAZ (pEF-Flag-TAZ) or with the empty vector (pEF-BOS). The cells were also transfected the expression vector for PPARγ (pSPORT-PPARγ) or empty vector as indicated and were exposed to either the vehicle or 1 µM rosiglitazone. Forty-eight hours after transfection, the cells were harvested and used for luminometry. Luciferase activities were normalized for transfection efficiency using *renilla* activity and all values were expressed as a fold induction relative to basal promoter activity. The results shown are representative of three independent experiments. (C) Primary calvarial cells from Col.1-TAZ (T) or WT (W) mice were treated with 150 ng/ml rhBMP-2 for 2 days and harvested for chromatin immunoprecipitation assays. Soluble chromatins were prepared and precipitated using control IgG or anti-Flag antibodies. The Runx2 binding site in the mouse osteocalcin promoter was amplified by PCR. (D–F) C3H10T1/2 cells were transiently cotransfected with BRE-Luc, p3TP-Lux, and TOPflash and with the expression vector for TAZ (pEF-Flag-TAZ) or an empty vector (pEF-BOS). Cells were exposed to the vehicle or BMP-2, TGF-β, or Wnt-3A CM for BRE-Luc, p3TP-Lux, and TOPflash, respectively. Luciferase activity was measured as described above. Data are expressed as means ± S.D., and the graphical results are representative of three independent experiments, each performed in triplicate (*p<0.05 vs. control).

TAZ has been shown to interact with many partners to control its target gene specificity, particularly through its function as a transcriptional coregulator [14]. In the case of osteoblastogenesis, TAZ functions as a coactivator of Runx2 while it acts as a corepressor of PPARγ. Thus, to understand the cellular mechanism behind the increased bone mass of Col.1–TAZ mice, we first tried to confirm previously reported signaling in vitro. Transfection of the p6OSE2-Luc plasmid, which contains 6 copies of the Runx2 binding element, into C3H10T1/2 cells resulted in basal promoter activity that was robustly increased by cotransfection of the pcDNA-til plasmid, as expected. Although cotransfection of pEF-Flag-TAZ in the absence of til does not affect the luciferase activity, cotransfection of both TAZ and til led to a significant increase in til-mediated activity by 112%, confirming that TAZ enhances Runx2-mediated signaling at the transcriptional level (Fig. 5A).

In addition, the induction of reporter activity from PPRE-Luc, which contains the PPARγ response element, by transfection of PPARγ (pSPORT-PPARγ) was significantly downregulated by cotransfection of the TAZ expression vector both in the absence and presence of rosiglitazone as expected (Fig. 5B). Furthermore, the ChIP assay using an osteocalcin promoter, showed that TAZ is engaged in Runx2 transcriptional complexes (Fig. 5C).

We next investigated the possibility that TAZ can affect osteoblast differentiation signaling pathways other than Runx2 signaling. To this end, we performed a series of promoter reporter assays that evaluate the activity of BMP-2, TGF-β, and Wnt signaling pathways by transient transfection of BRE-Luc, p3TP-Lux, or TOPflash reporter plasmids, respectively. As shown in Fig. 5D, the pEF-Flag-TAZ does not affect BMP-2 (100 ng/mL) mediated induction of BRE-Luc reporter activity. Interestingly however, overexpression of TAZ significantly increased p3TP-Lux activity, both in the absence and presence of TGF-β (5 nM) (Fig. 5E). In contrast, TOPflash activity, induced by Wnt-3A CM (0.5×), was significantly downregulated by cotransfection of pEF-Flag-TAZ (Fig. 5F).

### TAZ regulates TGF-β-mediated Smad translocation

**Figure 6:**
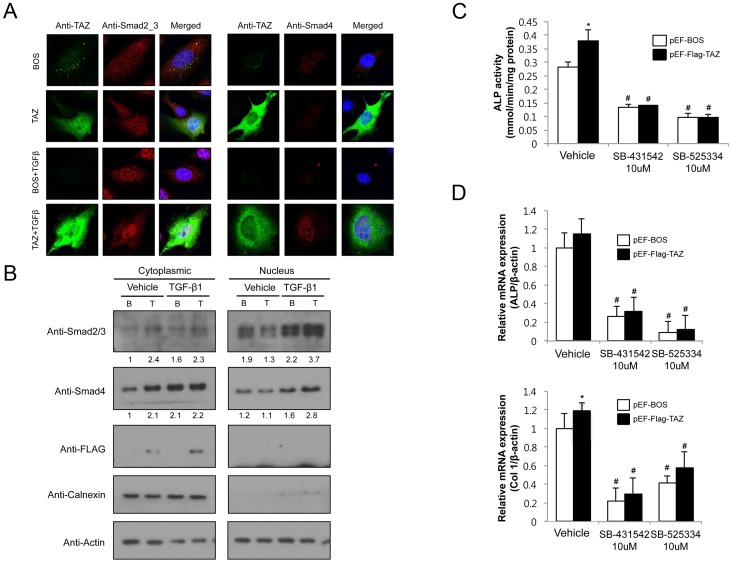
TAZ enhances TGF-β-mediated Smad nuclear accumulation. (A) The UAMS-32 cells were transfected with pEF-BOS (BOS) or pEF-Flag-TAZ (TAZ). Endogenous Smad2/3 and Smad4 localization was determined following treatment with the vehicle or TGF-β. (B) Sub-cellular localization of Smad2/3 and Smad4. UAMS-32 cells were transiently transfected with pEF-BOS or pEF-Flag-TAZ, fractionated into nuclear and cytosolic fractions, and analyzed by immunoblotting for Smad2/3, Smad4, and Flag. The purity of nuclear and cytosolic fractions was confirmed by immunoblotting for Calnexin. (C) C3H10T1/2 cells transfected with pEF-BOS or pEF-Flag-TAZ were cultured for 8 days in osteogenic medium (OM) including ascorbic acid and β-glycerophosphate in the absence or presence of TGF-β inhibitors (SB-431542 or SB-525334). ALP activity was measured in the cell layer and normalized to cellular protein content. Data are expressed as means ± SD (* p<0.05 vs. pEF-BOS, # p<0.01 vs. vehicle) (D) Quantitative RT-PCR analysis of Col 1 and ALP in C3H10T1/2 cells (* p<0.05 vs. pEF-BOS, # p<0.01 vs. vehicle).

Having identified that TAZ significantly upregulates TGF-β signaling, we next determined whether overexpression of TAZ affected the nuclear accumulation of Smads, which are essential to TGF-β signaling. As shown in Fig. 6A, Smad2/3 are distributed throughout the cytoplasm and nucleus of both pEF-BOS and pEF-Flag-TAZ transfected UAMS-32 cells in the unstimulated state. When we added TGF-β to these cells, Smad2/3 and Smad4 accumulated in the nucleus. Overexpression of TAZ in this setting has significantly increased the TGF-β–mediated nuclear localization of Smad2/3. The translocation of Smad4 also increased in a similar manner. Cell fractionation and Western blot analysis also confirmed that TGF-β-mediated nuclear accumulation of Smad2/3 and Smad4 was further increased by transfection of TAZ (Fig. 6B, right panel).

We next asked whether the increased TGF- β signaling by overexpression of TAZ is specifically associated with enhanced osteoblast differentiation. Towards this end, we first investigated the ALP activities in C3H10T1/2 cells, mouse mesenchymal cell line, in the absence or presence of soluble TGF- β inhibitors. As shown in Fig. 6C, transfection of C3H10T1/2 cells with TAZ expression vector has significantly increased the ALP activities by 35%. However, cotreatment with SB-431542 or SB-525334 has not only inhibited ALP activities compared with vehicle treatment but also abrogated the TAZ-mediated enhancement of ALP activities. In addition, treatment of C3H10T1/2 cells has significantly blunted the TAZ-mediated induction of osteoblast-specific genes including Col1 and ALP (Fig. 6D). Taken together, these results suggest that enhancement of TGF-β signaling, in addition to increased Runx2-mediated signaling, may contribute to the increased bone mass in Col.1-TAZ mice.

## Discussion

Using a collagen type 1 promoter-driven transgenic mice model, we demonstrate that osteoblast-specific overexpression of TAZ results in increased bone mass both *in vivo* and *ex vivo* with a reciprocal increase in adipogenesis in ex vivo culture of calvarial cells.

TAZ was initially identified for its ability to interact with 14-3-3 proteins in the cytoplasm and is sharing sequence homology with YAP [13]. TAZ interacts with a number of transcription factors and exhibits transcriptional regulatory functions [14]. In addition to regulating genes involved in embryogenesis [24], and development of bone [16], [18], muscle [25], fat [16], lung [26], heart, and limbs [27], TAZ also plays a role in transcriptional regulation of mesenchymal stem cell differentiation [16]. TAZ functions as a coactivator of Runx2 and corepresses PPARγ, thereby enhancing osteoblastogenesis at the expense of adipogenesis [28]. Deletion of TAZ in zebrafish results in a lack of ossification and ventral curvature [16]; however, TAZ null mice show only partial lethality and the mice that do survive exhibit a slightly shorter skeleton [20], [29]. Therefore, it would be an intriguing question whether osteoblast-specific overexpression of TAZ results in skeletal phenotype alterations in a mice model.

In this study, we have clearly provided evidence that osteoblast-specific overexpression of TAZ could increase bone formation in vivo. First, transgenic mice had an approximately 20∼26% increase in trabecular bone volume up to 32 weeks of age, indicating that overexpressed TAZ enhances bone mass gain at this growing phase. Second, histomorphometric analyses showed increased bone formation rates and osteoblast numbers. Third, at the cellular levels, calvarial cells from transgenic mice exhibited increased ALP activities. Finally, calvarial cells from TAZ mice showed increased expression of the osteoblast-specific transcription factors, Runx2 and osterix, and osteoblast marker genes including type 1 collagen, ALP, and osteocalcin. These results are consistent with previous studies that demonstrate an essential role of TAZ in osteoblastic differentiation of mesenchymal stem cells [30] and in *in vivo* bone formation in zebrafish [16]. Our results now identify that TAZ is also critical for mediating bone formation in a mammalian system and enhancing bone formation during the postnatal period.

Previous studies show that TAZ, as a transcriptional coactivator, interacts with Runx2 through its WW domain and regulates Runx2-dependent osteoblast differentiation [18]. In our hands, we have also confirmed that TAZ enhanced Runx2-mediated transcriptional activity of p6OSE2-Luc plasmid, which contains 6 copies of OSE2, indicating that the TAZ is specifically activating Runx2 activity at the OSE2 element. The lack of TAZ-mediated enhancement of reporter activity in the absence of til indicates that expression of Runx2 is required for the activity of TAZ in increasing the osteoblast differentiation.

Besides Runx2-mediated transcription, we were also able to demonstrate that TAZ enhanced TGF-β-mediated signaling. Indeed, our transient transfection study shows that TAZ enhances the TGF-β-mediated increase in 3TP-lux reporter activity. In addition, overexpression of TAZ results in nuclear accumulation of Smad 2 and 3, which allows them to regulate transcription of target genes by interacting with DNA-binding partners and transcriptional coactivators and corepressors [31]. Consistent with our study, Varelas et al. also demonstrated that TAZ plays a significant role in Smad nucleocytoplasmic shuttling in human embryonic stem cells. They show that TAZ binds to the heteromeric Smad2/3-4 complex in response to TGF-β and is then recruited to a TGF-β response element while Smad complex can not accumulate in the nucleus in the absence of TAZ [32]. The increased TGF-β signaling in response to TAZ seems to be specifically associated with enhanced osteoblast differentiation as the treatment with TGF-β inhibitor could effectively abrogated the TAZ-mediated osteoblast differentiation. Interestingly, in our study, transfection of TAZ did not significantly activate the transcriptional activity of the BRE-luc construct. Although the relative roles of BMP-2 and TGF-β in bone formation are controversial, BMP-2 and -4 are absent during the fracture healing process, but serum levels of TGF-β are dramatically increased [33] suggesting that BMPs and TGF-β play differential roles depending on the physiological or pathological conditions. Given that TGF-β plays a role in undifferentiated mesenchymal stem cell proliferation, osteoblast precursor recruiting, and bone matrix production during bone repair [34], our results suggest that the positive regulation of bone mass by TAZ may be in part mediated by enhancement TGF-β signaling.

TOPflash, the reporter for Wnt signaling, was significantly inhibited in response to TAZ overexpression. This result is interesting considering the critical role of Wnt/β-catenin signaling in osteoblast. However, our data are analogous with a recent study that demonstrated that TAZ has inhibitory effects on the Wnt/β-catenin pathway through physical interactions between TAZ and Dishevelled (DVL) in the cytoplasm [35]. This interaction between TAZ and Wnt/β-catenin pathways in the process of osteoblastic differentiation should be a subject of further research.

Notably, opposite to the positive effect on Runx2 and TGF-β signaling, we identified that TAZ significantly suppresses adipogenic differentiation of calvarial cells ex vivo, as a result of suppressed PPARγ signaling. This result corresponds with a previous study that demonstrates an inhibitory role of TAZ on adipocyte differentiation, on aP2 gene expression, and on PPARγ mediated transactivation in vitro [16] and again confirms the reciprocal regulation of differentiation to osteoblasts and adipocytes in mesenchymal stem cells.

In this study, Col.1-TAZ mice did not show alteration of bone resorption parameters. Although 2.3 kb Col 1 promoter drives transgenes specifically in mature osteoblasts [36], previous studies demonstrated that overexpression of Runx2 driven by 2.3 kb Col 1 promoter led to increased bone resorption phenotypes, primarily due to increased RANKL expression [37], [38]. A recent study also showed that treatment with small molecule that activates TAZ could protect ovariectomy-induced bone loss in vivo [39]. However, in our study, the RANKL/OPG expression levels in calvaria cells from Col.1-TAZ mice were not significantly different from those from WT mice. In addition, culture of bone marrow macrophages (BMMs) from WT and Col.1-TAZ mice in the presence of RANKL did not result in significant difference in the number of osteoclasts. Finally, the number of TRAP-positive osteoclasts in tibiae of Col1-TAZ was not significantly different from that in WT mice. These results suggest that Col 1 promoter-driven overexpression of TAZ does not significantly alter bone resorption parameters. We speculate that although TAZ overexpression leads to increased transcriptional activity of Runx2, the overall consequences of TAZ overexpression at the cellular and tissue level may not be identical to Runx2 overexpression, possibly through different downstream signaling with as yet identified molecular interactions.

In contrast to the high bone mass phenotype in male mice, there was no clear BMD change in female mice. The reason for this discrepancy is not known, but it is interesting to note that we recently observed similar results in Col.1-PPARγ mice [10]. In Col.1-PPARγ mice, the males exhibited low BMD compared to the WT, but no significant changes were noted in the female mice. However, female Col.1-PPARγ mice lose a greater amount of bone mass after ovariectomy indicating that estrogen may mask the effects of PPARγ overexpression, which becomes evident only after loss of estrogen [10]. Given that TAZ plays a role as a corepressor of PPARγ, it could be postulated that a similar mechanism might have resulted in the observed difference between male and female Col.1-TAZ mice. Another possibility might be the interaction between TAZ and estrogen receptor since TAZ has been shown to bind to WW-domain binding protein 2 (WBP2), which functions as a transcriptional coactivator of ER [40].

In conclusion, osteoblast-targeted overexpression of TAZ resulted in significant bone mass gain in a growing mouse model. Although TAZ is a transcriptional modulator of mesenchymal stem cell differentiation, our results suggest that TAZ also plays a role in bone mass regulation during the postnatal period, and therefore could be utilized as a potential therapeutic target for the treatment of osteoporosis.

## Supporting Information

Figure S1 **Body weight and bone phenotype in Col.1–TAZ mice line 23.** Total body BMD (A) was analyzed by dual energy X-ray absorptiometry (PixiMus). Body weight (B) was measured in male Col.1– TAZ mice and their WT littermates at 8, 12, 16, 20 and 32 weeks (* p<0.05, **p<0.01 vs. WT). (C) Static morphometric parameters including trabecular bone volume expressed as percentage of total tissue volume (BV/TV), trabecular number (Tb.N), trabecular thickness (Tb.Th), and trabecular spacing (Tb.Sp), cortical bone area (Ct.Ar) and average cortical thickness (Ct.Th) were measured with the CT images (* p<0.05, ** p<0.01 vs. WT littermates). Data are expressed as means ± SD.
(TIF)
