# Supplementary figures and images for: Osteoblast-Targeted Overexpression of TAZ Increases Bone Mass In Vivo
Source: PLoS One. 2013 Feb 18;8(2):e56585. doi: 10.1371/journal.pone.0056585 (PMC3575506; doi:10.1371/journal.pone.0056585)

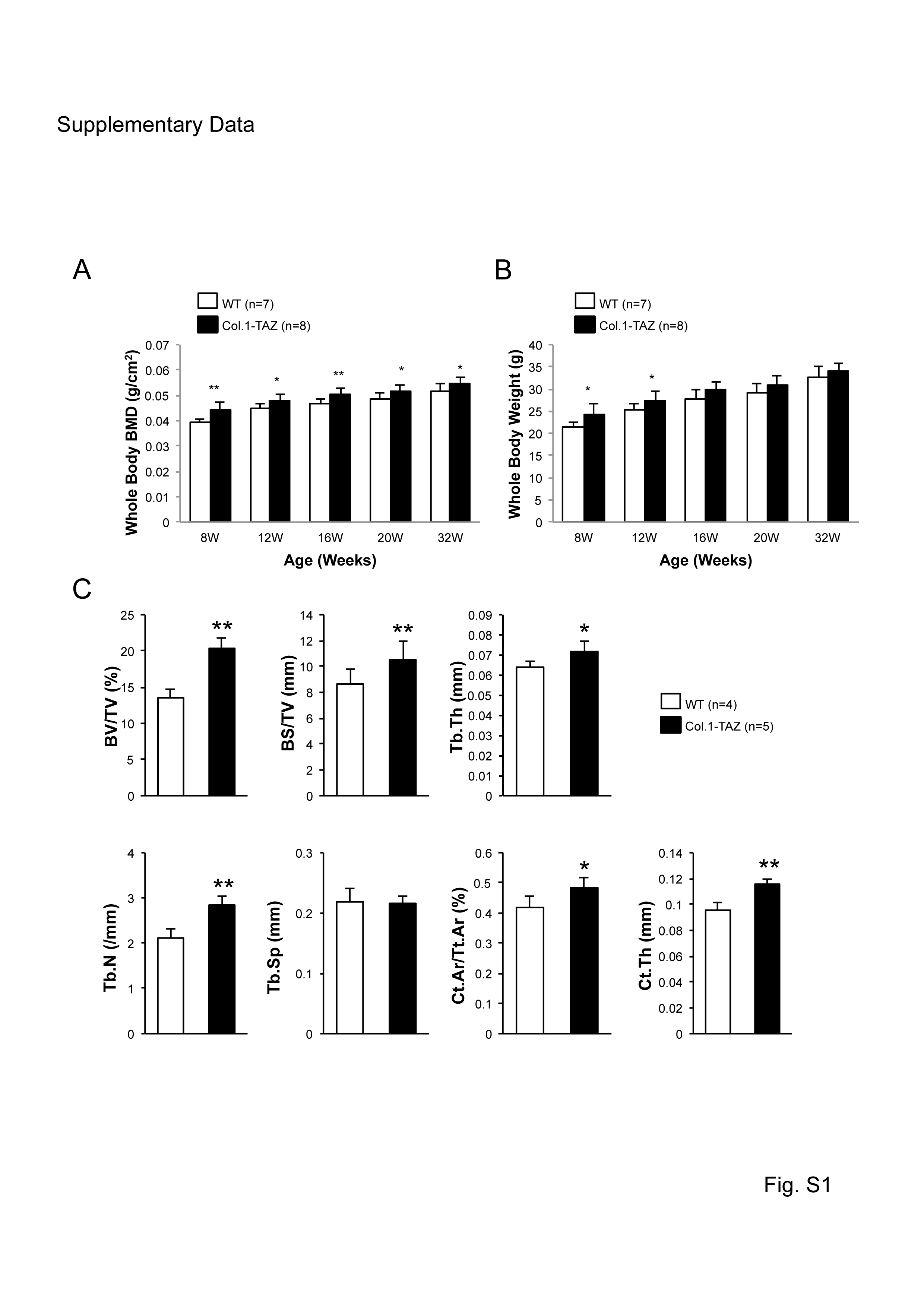

Supplement: Figure S1 — Body weight and bone phenotype in Col.1–TAZ mice line 23. Total body BMD (A) was analyzed by dual energy X-ray absorptiometry (PixiMus). Body weight (B) was measured in male Col.1– TAZ mice and their WT littermates at 8, 12, 16, 20 and 32 weeks (* p<0.05, **p<0.01 vs. WT). (C) Static morphometric parameters including trabecular bone volume expressed as percentage of total tissue volume (BV/TV), trabecular number (Tb.N), trabecular thickness (Tb.Th), and trabecular spacing (Tb.Sp), cortical bone area (Ct.Ar) and average cortical thickness (Ct.Th) were measured with the CT images (* p<0.05, ** p<0.01 vs. WT littermates). Data are expressed as means ± SD. (TIF) [file pone.0056585.s001.tif]
